# Special Issue Editorial: Current Advances in Liquid Crystals

**DOI:** 10.3390/molecules26123713

**Published:** 2021-06-18

**Authors:** Pradip K. Bhowmik

**Affiliations:** Department of Chemistry and Biochemistry, University of Nevada Las Vegas, 4505 S. Maryland Parkway Box 454003, Las Vegas, NV 89154-4003, USA; pradip.bhowmik@unlv.edu

The broad field of liquid crystals (LCs) has attracted the attention of chemists, physicists, biologists and engineers alike since the discovery of liquid crystalline phase by the Austrian botanist Friedrich Reinitzer in 1888 [1]. LC phases combine the molecule/macromolecular properties of crystals with the flow properties of liquids. As such, they have provided the foundation for a revolution in the low-power, flat-panel display technology commonly known as liquid crystal displays (LCDs). Many diversified, chemical–structural architectures that are rod-shaped, disc-shaped and bent-shaped, among others, exhibit various types of LC phases. These bewildering chemical structures include organic molecules, main-chain polymers, side-chain polymers, hyperbranched polymers, dendrimers, ionic molecules and even zwitter ionic molecules, to mention a few. They have many technological applications because of their chemical diversities and types of LC phases. These applications range from the structural materials to neural interface in biomedical devices. For notable examples, Kevlar—lyotropic LC aromatic polyamide—and Vectra—thermotropic aromatic LC polyester—are the basis of lightweight, high-strength materials for a number of civilian and military applications, including their important contributions in body armor. Side-chain LC polymers are known as functional polymers for non-linear optical properties and data storage capabilities [2,3]. Additionally, ionic liquid crystals (ILCs) that form columnar, smectic and bi-continuous cubic phases can provide well-organized 1D, 2D and 3D channels capable of transporting ions and electrons [4,5]. They can be used as electrolytes for batteries and photovoltaics, semiconductors and electroluminescence and electrochemical devices. ILCs are the recent addition to the ever-expanding field of LCs [6,7].

This Special Issue on Current Advances in Liquid Crystals aims at collecting some of the specialists working with liquid crystals, to shed light on the recent activities in the field. The papers cover many aspects of liquid crystals including H-bonded liquid crystals, optical textures and orientational structures in cholesteric droplets, ILCs and light-responsive LC copolymers, thus reflecting the many interests of the scientific community active in this field.

In reference [8], Alnoman et al. have investigated the synthesis, optical and geometrical approaches of new natural fatty acids’ esters/Schiff base liquid crystals that demonstrate the liquid crystals of smectogenic phases can be prepared from palmitic, oleic and linoleic all of which are natural fatty acids. The type and stability of LC phases depend on the length and conformation of the terminal alkenyl fatty acid chains. Computational calculations of different configurational isomers reveal that geometrical and thermal properties are influenced by the degree of unsaturation of the fatty acid alkenyl terminal chains.

In reference [9], Alhaddad et al. describe on the experimental and theoretical approaches of new nematogenic chair architectures of supramolecular H-bonded liquid crystals. These are based on intermolecular interactions of H-acceptors, 4-(2-(pyridin-4-yl)diazenyl-(2- or 3-)chlorophenyl) 4-alkoxybenzoates and H-donors, 4-n-alkoxybenzoic acids. All of them form nematic phases, however, the temperature range of nematic phases depends on the location and spatial orientation of lateral chlorine substituent present in the central ring of H-acceptor as well as the length of terminal chains. Computation calculations reveal that these complexes are in a chair form molecular geometry. 

In reference [10], Al-Mutabagani et al. describe on symmetrical U- and wavy-shaped supramolecular H-bonded systems; geometrical and mesomorphic approaches. These complexes containing seven phenyl rings are formed between 4-n-alkoxyphenylazobenzoic acids and 4-(2-pyrydin-3-yl)diazenyl)phenyl nicotinate. They exhibit enantiotropic SmC phases as established by the miscibility studies with a standard SmC compound. Theoretical calculation results reveal these LC complexes are in nonlinear geometry with U-shaped and wavy-shaped structures, however, wavy-shaped structures exhibit higher temperature range of LC phases than U-shaped structures. 

In reference [11], Alhaddad et al. also describe on chair- and V-shaped of H-bonded supramolecular complexes of azopheyl nicotinate derivatives; mesomorphic and DFT molecular geometry aspects. These complexes are prepared by the intermolecular interactions between 4-(4-(hexyloxy)phenylazo)methyl)phenyl nicotinate and 4-n-alkoxybenzoic acids. All of them exhibit enantiotropic nematic phases of alkoxy chain lengths studied. Theoretical calculation results reveal that these complexes are in nonlinear geometry with chair- and V-shaped geometry, however, chair-shaped complexes are found to exhibit longer range of mesophase than that of V-shaped complexes.

In reference [12], Alnoman et al. also describe on induced phases of new H-bonded supramolecular liquid crystal complexes; mesomorphic and geometrical estimation. These supramolecular complexes of five rings architectures are prepared from H-bonded interactions between 4-(2-(pyridin-4-yl)diazenyl-3-methylphenyl 4-alkozybenzoates and 4-n-alkoxyphenyliminobenzoic acids. These complexes exhibit SmC, SmA and nematic phases as determined experimentally and theoretically. Moreover, their geometric parameters are dependent on the electronic nature of the molecular shape and on the flexible chain lengths. 

In reference [13], Gardymova et al. describe on optical textures and orientational structures in cholesteric droplets with conical boundary conditions. The director configurations of cholesteric phase are identified by polarizing optical microscopy. The axisymmetric twisted axial-bipolar configuration with the surface circular defect at the droplet’s equator is formed at the relative chirality parameter N_0_ ≤ 2.9. The intermediate director configuration with the deformed circular defect is formed at 2.9 < N_0_ < 3.95, the layer-like structure with the twisted surface defect loop is found at N_0_ ≥ 3.95. These results have practical implications in electrically controlled shutters, sensors, lasers and polarizers.

In reference [14], Bhowmik et al. describe on thermotropic liquid-crystalline and light-emitting properties of bis(4-alkoxyphenyl) viologen bis(triflimide) salts. Even though triflimide salts are not conducive to ILCs because of their large size counterion, the studied salts containing triflimide counterions exhibit thermotropic LC properties that depends on the length of alkoxy chains. The salts with short alkoxy salts do not form LC properties, those with intermediate length of alkoxy chains show both crystal-to-LC transitions (T_m_s) and LC-to isotropic transitions (T_i_s), and those with long alkoxy chains show T_m_s only after which they form SmA phases that persist up to the decomposition at high temperatures. 

Finally, Alauddin et al. report on liquid crystalline copolymers containing sulfonic and light-responsive groups: from molecular design to conductivity in reference [15]. By using a cascade of reversible addition-fragmentation chain polymerization reaction, they have tailored different side-chain polymeric structures by controlling monomer microstructures and configurations. The resulting polymers show simultaneous liquid crystalline properties and appreciable conductivity with low concentration of polar sulfonic acid groups. Their light-responsiveness via reversible trans-to-cis photoisomerization of azobenzene groups and the local activation of conductivity at relatively low temperatures open the possibility for their use as polymer electrolytes for energy conversion and storage. 

To conclude, I believe that this Special Issue on Current Advances in Liquid Crystals touches on the latest advancement in several aspects related to liquid crystals including H-bonded liquid crystals, optical textures and orientational structures in cholesteric droplets, ILCs and light-responsive LC copolymers. I wish to express my deepest and sincere gratitude to all authors who contributed, for having submitted manuscripts of such excellent quality. I also wish to thank the Editorial Office of Molecules for the fast and professional handling of the manuscripts during the whole submission process and for the invaluable help provided. 
